# Association between heat stress and oxidative stress in poultry; mitochondrial dysfunction and dietary interventions with phytochemicals

**DOI:** 10.1186/s40104-016-0097-5

**Published:** 2016-06-28

**Authors:** Abdollah Akbarian, Joris Michiels, Jeroen Degroote, Maryam Majdeddin, Abolghasem Golian, Stefaan De Smet

**Affiliations:** Department of Animal Production, Laboratory for Animal Nutrition and Animal Product Quality, Ghent University, Proefhoevestraat 10, Melle, 9090 Belgium; Centre of Excellence in the Animal Science Department, Ferdowsi University of Mashhad, P.O. Box: 91775–1163, Mashhad, Iran; Department of Applied Biosciences, Ghent University, Valentin Vaerwyckweg 1, Ghent, 9000 Belgium

**Keywords:** Antioxidant enzymes, Avian uncoupling protein, Electron transport chain, Flavonoids, Heat Stress, Mitochondrion, Oxidative stress, Poultry

## Abstract

Heat as a stressor of poultry has been studied extensively for many decades; it affects poultry production on a worldwide basis and has significant impact on well-being and production. More recently, the involvement of heat stress in inducing oxidative stress has received much interest. Oxidative stress is defined as the presence of reactive species in excess of the available antioxidant capacity of animal cells. Reactive species can modify several biologically cellular macromolecules and can interfere with cell signaling pathways. Furthermore, during the last decade, there has been an ever-increasing interest in the use of a wide array of natural feed-delivered phytochemicals that have potential antioxidant properties for poultry. In light of this, the current review aims to (1) summarize the mechanisms through which heat stress triggers excessive superoxide radical production in the mitochondrion and progresses into oxidative stress, (2) illustrate that this pathophysiology is dependent on the intensity and duration of heat stress, (3) present different nutritional strategies for mitigation of mitochondrial dysfunction, with particular focus on antioxidant phytochemicals.

Oxidative stress that occurs with heat exposure can be manifest in all parts of the body; however, mitochondrial dysfunction underlies oxidative stress. In the initial phase of acute heat stress, mitochondrial substrate oxidation and electron transport chain activity are increased resulting in excessive superoxide production. During the later stage of acute heat stress, down-regulation of avian uncoupling protein worsens the oxidative stress situation causing mitochondrial dysfunction and tissue damage. Typically, antioxidant enzyme activities are upregulated. Chronic heat stress, however, leads to downsizing of mitochondrial metabolic oxidative capacity, up-regulation of avian uncoupling protein, a clear alteration in the pattern of antioxidant enzyme activities, and depletion of antioxidant reserves.

Some phytochemicals, such as various types of flavonoids and related compounds, were shown to be beneficial in chronic heat-stressed poultry, but were less or not effective in non-heat-stressed counterparts. This supports the contention that antioxidant phytochemicals have potential under challenging conditions. Though substantial progress has been made in our understanding of the association between heat stress and oxidative stress, the means by which phytochemicals can alleviate oxidative stress have been sparsely explored.

**Electronic supplementary material:**

The online version of this article (doi:10.1186/s40104-016-0097-5) contains supplementary material, which is available to authorized users.

## Background

Heat exposure affects poultry production on a worldwide basis and has a significant impact on well-being and production. Heat stress (HS) occurs when the amount of heat produced by an animal surpasses the animal’s capacity to dissipate the heat to its surrounding environment. This imbalance may be caused by variations in a combination of environmental factors (e.g. sunlight, thermal irradiation, air temperature, humidity, and movement) and characteristics of the animal (e.g. species, gender, and rate of metabolism) [1]. Animals experiencing HS tend to reduce their heat production by limiting feed intake, with subsequent negative effects on growth performance. Therefore, HS has been a great concern among scientists and poultry producers for many decades, particularly in arid (dry, hot all year) and in tropical (wet, hot all year) regions of the world, as well as in other climates due to surges in temperature during the spring and summer months. Two major categories of HS, i.e. “acute HS” and “chronic HS” can be distinguished. Acute HS refers to a short and rapid rise in ambient temperature. Chronic HS refers to a high ambient temperature over a long period of time (days to weeks), permitting acclimazation to the environment. Further, chronic HS is categorized as either “cyclic chronic HS,” which refers to a limited period of heat exposure followed by comfortable temperature for the rest of the day, or as “constant chronic HS” whereby the animal is continuously confronted with a high ambient temperature. The physiological consequences of HS are numerous and have been reviewed in the past [1–4]. These physiological alterations can be summarized as: increased body core temperature, reduced voluntary feed intake, depressed immunity, alteration of the electrolyte balance and blood pH, impairment in endocrine and reproductive functions, decreased energy availability to cells, alteration in the digestibility and metabolism of various nutrients, disruption in the structure and function of intestinal epithelium, alteration of the normal and protective microbiota, and increased circulatory cortisol and corticosterone levels. A number of mitigation strategies have been proposed and the reader is referred to some concluding reviews [1–6].

More recently, the involvement of HS as an inducer of oxidative stress (OS) has been acknowledged [7–10]. Oxidative stress is defined as the presence of reactive species (RS) in excess of the available antioxidant capacity of animal cells [11]. Many radicals and metabolic substances are described as potentially toxic and are defined as “reactive oxygen/nitrogen/chlorine species” [11]. These substances are highly reactive and can modify several biologically cellular macromolecules, such as proteins, lipids, and nucleic acids (DNA and RNA) [12]. These phenomena contribute to the development of several metabolic dysfunctions, including cell death by causing “oxidative stress” and “oxidative damage” [11]. Oxidized molecules abstract electrons from other molecules, resulting in a chain reaction and, if not controlled, this reaction can cause extensive tissue damage. In addition, OS may alter the redox equilibrium of several cellular redox couples [e.g. reduced glutathione (GSH) and glutathione disulfide (GSSG) and reduced and oxidized thioredoxin] leading to an altered expression of key enzymes in detoxification, antioxidant defense, cell transitions, inflammatory responses, etc.. Cells have evolved defense systems to control the production of RS. These include both non-enzymatic low-molecular weight (e.g. vitamin C, GSH, and uric acid) and enzymatic high-molecular weight [e.g. superoxide dismutase (SOD), glutathione peroxidase (GSH-Px), and arylesterase] compounds. They limit the rate and progression of oxidation and thereby protect cells from oxidative damage. Some low-weight molecular antioxidants are exclusively food derived, e.g. tocopherols. Finally, upon damage, repair mechanisms will be activated.

## Oxidative stress is associated with heat stress

### Heat stress induces mitochondrial dysfunction

**Fig. 1:**
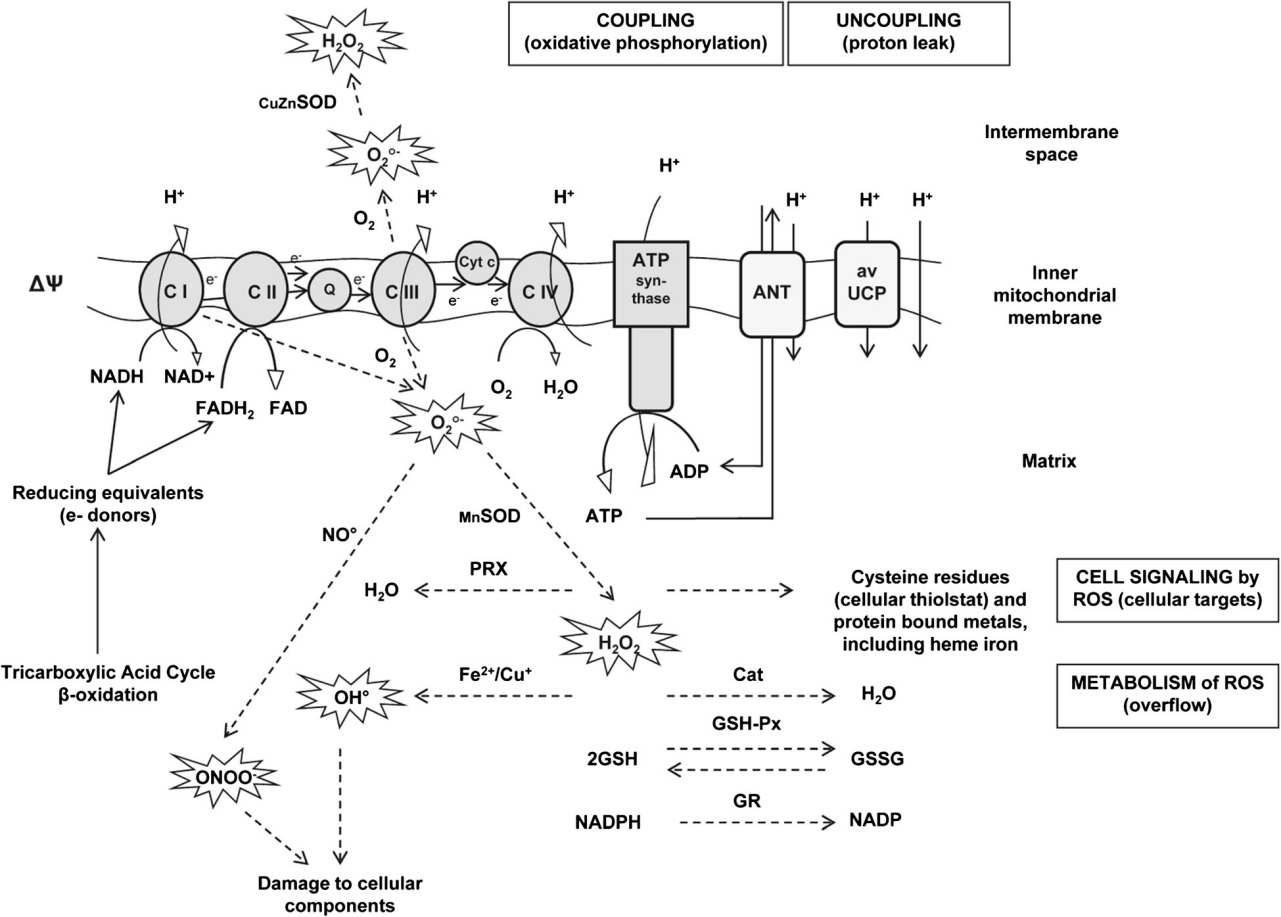
Mitochondrial energy transduction and patho-physiology of oxidative stress upon heat stress. Mitochondrial electron transport chain (ETC.; C I, complex I; C II, complex II; Q, coenzyme Q; C III, complex III; cyt c, cytochrome c; C IV, complex IV), ATP synthase (coupling through oxidative phosphorilation) and uncoupling mechanisms (non-protein or protein, by avian uncoupling protein, avUCP or A nucleotide translocator, ANT, catalyzed proton leak) are shown. In the initial phase of acute HS the mitochondrial substrate oxidation (tricarboxylic acid cycle and/or β-oxidation) and ETC. activity are increased resulting in more reduced state of the electron carriers of the ETC. and an increase of ΔѰ resulting in elevated superoxide production (O_2_°^−^), while during the later stage of acute HS, downregulation of avUCP worsens the oxidative stress. Chronic HS, however, leads to downsizing the mitochondrial metabolic oxidative capacity, upregulation of avUCP and a clear alteration in the pattern of antioxidant enzyme activities. Superoxide is readily dismutated by superoxide dismutase, SOD (CuZnSOD in intermembrane space and MnSOD in matrix) to give hydrogen peroxide (H_2_O_2_). H_2_O_2_ functions as the common ROS messenger in cell signaling due to its constant production, relative stability and diffusion properties. The primary targets of ROS for cell signaling are cysteine residues and protein bound metals, including heme iron. In cell signaling, downstream cascades will effect the activity of transcription factors of which AP-1, NF-kB and Nrf2 have been shown to be affected under HS conditions in poultry. Upon oxidative stress, an overflow of H_2_O_2_ can either be controlled by catalase (CAT; low affinity, high reactivity) and/or the glutathione-peroxidase/glutathione system (GSH-Px/GSH; high affinity, low reactivity) or undergo further reduction to yield the extremely reactive and dangerous hydroxyl radical (OH°) (Fenton reaction), possibly causing major damage to cellular biomolecules

Most cellular energy is produced through oxidative phosphorylation in mitochondria. The process of energy transduction requires the orchestrated action of four major respiratory enzyme complexes (Fig. 1). In normal physiological conditions, an estimated 1-2 % [12] to 2-4 % [13] of the total oxygen consumed during electron transport is not reduced to water by cytochrome c oxidase (complex IV), but rather to superoxide due to electron leakage in complexes I and III of the respiratory chain. Cells are equipped with efficient dismutation pathways; i.e. CuZnSOD acts in the intermembrane space (also present in cytosol) and MnSOD acts in the matrix to reduce superoxide to hydrogen peroxide (H_2_O_2_). In the presence of ferrous and cuprous ions, H_2_O_2_ can be further reduced to the extremely reactive and dangerous hydroxyl radical (OH°; Fenton reaction). In contrast to O_2_°^−^ and OH°, H_2_O_2_ can diffuse into cytosol via membranes, leading to new molecular reactions away from the production site and so reach new cells and tissues. The transmission to neighbouring cells and tissues can occur extremely quickly through several centimeters of tissue in a fraction of a second [12]. It is believed that under normal physiological conditions this reactive oxygen species (ROS) generation is not ultimately harmful and is securely controlled by the antioxidant defense mechanisms [14]. Furthermore, a mild production of ROS is crucial for proper cell function through its signaling actions. Due to its diffusion capacities and higher stability, H_2_O_2_ is the major ROS acting as a second messenger. In this respect, evidence shows that 2-Cys peroxiredoxin (2-Cys PRX; thioredoxin dependent for regeneration) functions as a regulator in controlling basal H_2_O_2_ levels in cells by its peroxidase activity (The floodgate hypothesis; [15]).

Although evidence for oxidative stress in heat stressed poultry has been published before, the molecular mechanisms were first described by Toyomizu and co-workers (Graduate School of Agricultural Science, Tohoku University; Sendai, Japan; e.g. Mujahid et al. [9]). The first step in the patho-physiology of HS appears to be an increase in cellular energy demand. Yang et al. [16] noted that promptly after acute heat exposure, cells increase their energy expenditure by 2-fold above normal levels. Mujahid et al. [17] showed that mitochondrial transportation and β-oxidation of fatty acids were enhanced after 6 h of acute HS. The latter was associated with elevated blood levels of non-esterified fatty acids. To meet the enhanced energy demand of cells and mitochondrial biogenesis, the production of reducing equivalents and the enzymatic activity of subunits of respiratory chain complexes are increased. Under physiological conditions, electron transport is tightly coupled to oxidative phosphorylation. This means that electrons do not usually flow through the electron transport chain (ETC.) to O_2_ unless ADP is simultaneously phosphorylated to ATP. Increased mitochondrial energy generation is intrinsically associated with an increase in ROS, owing to the fact that ROS production is favored by: 1) high levels of reduction of the electron carriers, particularly the coenzyme Q pool, 2) a large membrane potential (ΔѰ), and 3) high mitochondrial oxygen concentrations [18]. With regard to the first, the rate of mitochondrial ROS generation strongly increases with sigmoidal kinetics when the NADH/NAD^+^ ratio is increased. An increase in the NADH/NAD^+^ ratio dramatically elevates the degree of reduction of complex I reducing its capacity to accept electrons [14]. The increased production of reducing equivalents promotes electron transport in ETC., hence elevating ΔѰ. ΔѰ is the difference in electric potential between the matrix and mitochondrial intermembrane space and is essential to the action of ATP synthase. According to Kikusato and Toyomizu [19], mitochondrial ROS production is exponentially correlated with elevated ΔѰ. They found that, in heat stressed broilers, a 2.7 % or 5.3 mV increase in ΔѰ in respiratory state IV was accompanied by a 47 % increase in ROS production. This means that a slight increase of ΔѰ can have a substantial effect on ROS generation. Kikusato et al. [20] found that increased substrate oxidation (ETC. activity) is primarily responsible for the elevated ΔѰ in state IV; however, they were unable to assess whether a decrease in “proton leak” [lower uncoupling through down-regulation of avian uncoupling protein (avUCP); see below] happened. A sharp elevation of ΔѰ can reduce the efficiency of electron transfer which in turn leads to further leakage of electrons and eventually results in higher superoxide anion formation [21]. Further, it was shown that increased ROS production is independent from substrate supply, indicating that electron leakage and superoxide formation occur in several respiratory complexes [22, 23]. In addition, Kikusato and Toyomizu [19] postulated that the overproduction of ROS in heat stressed birds, when succinate was used as a substrate (yielding NADH), may be caused by reverse electron flow (and leakage) from coenzyme Q to complex I. In contrast, the cause of ROS overproduction upon glutamate oxidation (yielding flavin adenine dinucleotide; FADH2) remained unclear. Tan et al. [24] found that after 3 h of acute HS, the respiratory enzymes nicotinamide adenine dinucleotide cytochrome c reductase (NCCR; complex I) and cytochrome c oxidase (CCO; complex IV) showed a temperature-dependent (range of 32 ° to 38 °C) decrease in activity. This was not so for succinate cytochrome c reductase (SCCR; complex II). Azad et al. [25] observed that in chronic heat exposed chickens, the elevated ΔѰ and increased ROS production was found until d9 and d5 of HS and not thereafter, meaning that chickens became acclimatized to the stressor. Associated with this acclimation was a decrease in mitochondrial metabolic capacity evidenced by the decrease in 3HADH (3-hydroxyacyl CoA dehydrogenase, a key enzyme in the fatty acid β-oxidation) and CS [citrate synthase, an enzyme in the tricarboxylic acid cycle (TCA) and an indicator of aerobic metabolism] activity.

### Role of uncoupling proteins

Mitochondria have internal mechanisms to control superoxide production [14]. Uncoupling proteins (UCP) are mitochondrial inner membrane proteins belonging to the family of mitochondrial transporter proteins. Mild uncoupling by UCP, i.e. transferring protons back into the matrix, lowers ΔѰ and slightly stimulates electron transport. The complexes become more oxidized and the local concentration of oxygen is reduced. To date, the regulation of UCP remains under discussion. Echtay et al. [26] proposed a simple feedback mechanism in which superoxide is thought to peroxidize membrane phospholipids, forming hydroxynonenal, amongst other lipid peroxidation products, enhancing the action of UCP and adenine nucleotide translocator (ANT). The result is an enhancement of proton backflow. This feedback response limits mitochondrial ROS production upon OS. The picture in poultry is largely unknown. It is conceivable to state that down-regulation of avUCP, hampering mild-uncoupling in heat stressed birds, is a major contributor to the overproduction of ROS. Indeed, it was repeatedly shown that acute HS down-regulates the avUCP mRNA expression and protein level in skeletal muscle mitochondria (e.g. [22, 23]). The group of Toyomizu and co-workers showed later that mRNA levels declined after 6 h of acute HS, while protein levels were significantly lower only for the 12 h and 18 h measurements [17]. The same study also revealed that, contrary to avUCP, ANT transcript was not affected by HS. Both avUCP and ANT are known to be up-regulated (uncoupling activity; redox energy is dissipated as heat instead of being used for ATP synthesis) in cold stressed birds and thus play a role in thermogenesis. Apparently, only avUCP is implicated in the HS response (down-regulation of avUCP).

### Tissue damage

Overproduction of ROS in mitochondria can damage proteins, lipids, and DNA, further reducing energy generation efficiency and increasing ROS production in mitochondria. When HS is more severe and/or prolonged than what is justifiable for mitochondrial homeostasis, this can lead to decreased ATP synthesis, cellular calcium dyshomeostasis, and induction of the mitochondrial permeability transition, all of which predispose cells to apoptosis or necrosis. Evidence for mitochondrial damage following HS has been shown by many authors (see below). For example; Mujahid et al. [27] found that in 18 h heat stressed chickens, plasma and mitochondrial malondialdehyde (MDA) was 2.1- and 2.7-fold higher. They also found that 82 mitochondrial proteins were oxidized when compared with control chickens (non-heat-stressed). Mitochondria are particularly vulnerable to oxidative attack because their membranes are rich in poly-unsaturated fatty acids and proteins. In addition, OS will impact on the cellular redox environment and interfere with the oxidation/reduction potential of reactive thiols in proteins. The activity and regulation of signaling pathways and transcription factors [e.g. activator protein 1 (AP-1), nuclear factor kappa-light-chain-enhancer of activated B (NF-κB) and nuclear factor (erythroid-derived 2)-like 2 (Nrf2); see below] can be affected. It is believed that this series of events is the onset for cellular and tissue damage, causing major physiological and behavioral responses in birds which contribute to reduced productivity.

### Effect on cell signaling

The complex molecular communication network underlying cell biochemistry and function includes numerous components, kinases, phosphatases, and transcription factors, that have been conclusively proven to be sensitive to cellular and tissue redox changes [28]. ROS, whose constitutive generation in cells can thus be amplified in the context of HS, are important triggers and modulators of cell signaling and consequently cell behavior. Several redox-sensitive downstream pathways culminate in the activation of transcription factors, of which AP-1, NF-κB, and Nrf2 have been shown to be affected under HS conditions in poultry. AP-1 acts as a modulator of the expression of various genes in response to biological and environmental stimuli such as OS (phase II antioxidant defense such as glutathione S-transferase and NAD(P)H oxidoreductase), whereas NF-κB is principally involved in pro-inflammatory responses. Both transcription factors are involved in cell survival, differentiation, and growth. Upon activation, Nrf2 can bind specifically to the antioxidant response element (ARE) of the genome, which is located in the promoter region of stress-induced genes. Nrf2 induces the initiation of phase I and II enzymes. Linked with HS in poultry, cyclic chronic HS (35-day-old Japanese quails were exposed for 12 weeks to 34 °C for 8 h/d followed by 22 °C) has been shown to increase hepatic expression of cyclooxygenase-2, heat-shock proteins (HSP), AP-1 components (c-Jun and c-Fos), and NF-κB, and to reduce expression of Nrf2 [29, 30]. The latter corroborated with decreased antioxidant enzyme activities. Nevertheless, from other studies it appears that the exact outcome of the (de)activation of transcription factors during HS is hard to understand. For example, it has been shown that acute HS in pigs led to oxidative damage and up-regulation of antioxidant enzymes in red semitendinosus fibres (oxidative muscle; high mitochondrial density), but did not trigger a pro-inflammatory response, suggesting that the up-regulation of NF-κB-dependent genes upon HS is more complex and highly dependent on the stressor [31]. Hence, a better understanding of the impact of different levels of HS on cell signaling warrants attention; not in the least because many antioxidant phytochemicals appear to interact with cell signaling cascades and/or with transcription factors directly (e.g. [29]). In the context of HS, the heat-shock response needs discussion as well. Following increases in body temperature, cellular proteins may become unfolded and cell functions be compromized. The heat-shock response, through activation of heat-shock transcription factors and the elevated expression of inducible heat-shock proteins (HSP) and molecular chaperones, protects the cell against the accumulation of non-native proteins. HSP interact with those proteins to hold them properly together and retain them functional, hence exhibiting a major contribution to cell integrity under these stress conditions. It has been documented that exposing poultry to HS increases the concentrations of HSP70 and HSP90 [30, 32, 33]. Barret et al. [34] stated that ETC. dysfunction activates a heat-shock protein response, and that this response is mediated by OS. Inhibition of HSP70 expression in intestinal mucosa by quercetin led to a more severe extent of stress, as there was a significant elevation of serum corticosterone and heterophil:lymphocyte ratio, and a reduction in the enzymatic antioxidant response in acute HS chickens [33]. Pamplona and Costantini [14] outlined that quantification of stress response mediated by HSP is a promising biomarker to evaluate how an organism can maintain cellular functioning under environmental challenges and protein denaturing conditions. In this regard, Gu et al. [33] showed nicely that in acutely heat stressed broilers a higher HSP70 response correlated with a better antioxidant defence.

## Oxidative stress upon acute and chronic heat stress

A literature survey was performed and all studies that included a negative control (non-heat-stressed birds for comparison) were selected. Full details can be found in Additional file 1: Table S1 and Additional file 2: Table S2, addressing studies that investigated the effects of acute and chronic HS on the oxidative status of poultry, respectively (references included in Supplementary Material S1 and S2). Elevated levels of lipid peroxidation products (aldehydes, hydrocarbons, and isoprostanes) and protein carbonyls in serum, plasma, and tissues have been recognized as an index of OS. The elevation of MDA does not occur to the same extent in acute and chronic HS. For example, acute HS (30-day-old broilers were exposed for 5 h to 40 ± 1 °C; [35]) exhibited a 4-fold increase in MDA, whereas chronic HS (14-day-old broilers were exposed for 14 d to a cyclic chronic HS at 32 °C for 8 h/d followed by 24 °C; [36]) showed a 1.2- to 1.5-fold increment in MDA. Depending on the severity and duration of HS, the antioxidant system and associated enzymes behave differently. Typically, after acute HS, the activities of antioxidant enzymes (CAT, GSH-Px, and SOD) are increased sharply to protect cells against surplus superoxide formation. On the other hand, inconsistent results have been reported in chronic HS. Pamok et al. [37] reported that exposing 28-day-old broilers for 21 d to a constant chronic HS at 38 ± 2 °C leads to an increase in GSH-Px activity in erythrocytes until d11 of HS, while Seven et al. [38] found decreased GSH-Px activity in blood, liver, kidney, and heart from constant chronic HS exposed broilers (1-day-old, at 34 °C for 41 d). Differences in antioxidant enzyme activities might largely depend on the HS conditions, species, and tissue. Interestingly, Sahin et al. [29] found that, in chronic heat stressed quail, a reduced hepatic SOD, GSH-Px, and CAT activity corroborated with a reduction in Nrf2 expression. Another more clear difference exists between the acute and chronic HS response. Acute HS down-regulates avUCP synthesis leading to overproduction of ROS, whereas chronic HS works in the opposite way and up-regulates UCPs synthesis in an attempt to lower ROS production. Down-regulating avUCP and mRNA abundance upon acute HS occurred at all times until 18 h after starting acute HS [17]. Dridi et al. ([39]; 28-day-old ISA JV broilers were exposed for 10 d to 32 °C) and Azad et al. ([36]; 14-day-old Ross broilers were exposed for 14 d to constant chronic HS at 32 °C or 34 °C or to cyclic chronic HS at 32 °C for 8 h/d followed by 24 °C) showed that chronic HS enhanced avUCP transcript levels in pectoralis superficial muscle mitochondria by 71 % and 100 %, respectively. It has been reported that acute HS (16-day-old Cobb broilers were exposed for 18 h to 34 °C; [17]) increases the activity of 3HADH and CS, thereupon, stimulating metabolic oxidation capacity in mitochondria, leading to higher levels of mitochondrial superoxide, hence inducing OS. These increments were time-dependent; only after 6 h of HS was the activity of these enzymes significantly enhanced. After 12 and 18 h of HS, they were reduced to the levels of those of a non-heated group. On the contrary, Azad et al. [36] showed that exposing 14-day-old Ross broilers for 14 d to either of two constant chronic HS situations at 32 °C or 34 °C or to cyclic chronic HS at 32 °C for 8 h/d followed by 24 °C, decreased the activities of 3HADH and CS.

A number of chronic HS studies have been executed at the laboratory of Sahin and co-workers (Additional file 2: Table S2), all of which show higher MDA concentrations and lower antioxidant components (e.g. vitamins C, E, A, and co-factors Zn, Mn, Cu, Se) in tissues of broilers and Japanese quails. For instance, Sahin et al. [40] showed that chronic exposure to high temperature (10-day-old Japanese quails were exposed to 34 °C for 8 h/d followed by 22 °C for 32 d) decreased serum ascorbic and tocopherol concentrations by 40 % and 29 %. Based on their reports, following chronic HS, components of the animal’s antioxidant system became depleted. This supports the contention that the animal’s antioxidant system needs to be re-enforced upon chronic HS. Further, Gursu et al. [41] reported that HS (10-day-old Japanese quails were exposed to cyclic chronic HS at 34 °C for 8 h/d for 30 d) reduces the activity of arylesterase and paraoxonase in serum and that antioxidant supplementation (vitamin C and folic acid) inverts this reduction. Reducing the activity of arylesterase and paraoxonase following a shorter time of chronic HS has also been reported (22-day-old Hubbard broilers were exposed to cyclic chronic HS at 35 ± 1 °C for 8 h/d for 20 d, [42]. Gursu et al. [41] noted that these enzymes show free radical scavenging activity. Furthermore, it was found that serum homocysteine increases and folic acid and vitamin B12 decreases in chronic HS (e.g. [8, 43]), indicating enhanced conversion of methionine to cysteine to support GSH replenishment. In these reports, basal serum homocysteine levels ranged between 9.5 and 17 μmol/L and increased to levels between 21.1 and 24.0 μmol/L in heat stressed Japanese quails (increase between +41 % to +122 %). Two reports indicated an increase in GSH in the liver, the main site for GSH synthesis. Ramnath et al. [44] found an increase during 5 to 10 d of cyclic chronic heat stressed male Gramapriya chickens (28-day-old, at 40 ± 1 °C for 4 h/d, RH = 80 % ± 5 %). Willemsen et al. [45] showed higher hepatic total GSH levels and lower GSH/GSSG ratios in 4 week constant heat stressed Ross male broilers (14-day-old, 32 °C). These authors also found that plasma uric acid levels were increased, which corroborates with the result of Azad et al. [36]. It appears that birds adapt to chronic HS by up-regulation of levels of endogenous antioxidants such as GSH and uric acid, in line with what has been shown in other stress models (e.g. [46]; free-ranging in combination with severe feed restriction).

## Potential of antioxidants towards preventing/reducing oxidative stress in heat stressed poultry

### Strategies to reduce mitochondrial dysfunction in heat stressed broilers

Based on the mechanisms leading to mitochondrial dysfunction outlined above, 3 approaches are suggested here in order to deal with mitochondrial dysfunction in heat stressed birds; i.e. 1) reduce the membrane potential (e.g. mild uncoupling by using uncouplers or up-regulating avUCP), 2) increase ETC. efficiency (e.g. supplementation with coenzyme Q), or 3) enhance ROS detoxifying capacity (e.g. SOD activity, antioxidants acting either directly or indirectly through the activation of cell signaling pathways). The first strategy might appear ambigous. If the aim is to reduce the membrane potential through uncoupling, then substrates are oxidized producing heat instead of usable energy in the form of ATP. This might seem contra productive because: 1) this heat production may further enhance hyperthermia and 2) inefficient use of ingested feed may accentuate one of the key issues in heat stressed poultry, i.e. the reduced feed intake. Conversely, Azad et al. [47] found that using a mild uncoupler [cashew nut shell liquid (CNSL) as a source of anacardic acid] in chronic heat stressed broilers, increased feed consumption and weight gain and argued that this uncoupling might trigger enhanced feed intake. Although CNSL was not able to mitigate muscle MDA and H_2_O_2_ formation in the study of Azad et al. [47], it showed a mild-uncoupling effect in mitochondria in the study of Toyomizu et al. [48]. It remains equivocal whether selective uncoupling could, perhaps temporarily, relieve heat stressed poultry from OS upon short episodes of high environmental temperatures.

Coenzyme Q (also called ubiquinone) has received extensive interest in the scientific community. It acts both as an electron carrier in the ETC. and as an oil-soluble antioxidant within membranes and other lipophilic matrices. Coenzyme Q plays a crucial role in the ETC. since it functions as an electron carrier from complex I and II to complex III. Its antioxidant function is widely acknowledged; it inhibits both the initiation and propagation of lipid and protein oxidation and regenerates other antioxidants such as vitamin E. It is conceivable to assume that an increase in the bioavailability of coenzyme Q should be beneficial to heat stressed broilers. Coenzyme Q is biosynthesized in the body and can be obtained from food, in particular meat, fish, and vegetable oils are rich sources. It is assumed that biosynthesis is the major source. Broiler diets fortified with coenzyme Q have been shown to reduce the incidence of ascites (pulmonary hypertension syndrome) and the mortality of broilers [49], and this was attributed to improved ETC. efficiency upstream of coenzyme Q and higher hepatic mitochondrial antioxidant capabilities. Similar to heat stress, ascites has been shown to be associated with mitochondrial dysfunction [50, 51]. Enhanced performance upon coenzyme Q supplementation was also found in broilers under high altitude induced hypoxia [52].. Similarly, the role of L-carnitine should be recalled. L-carnitine plays a key role in energy metabolism of cells, mainly, by transferring acyl groups from cytoplasm to mitochondrial matrix for β-oxidation [53]. Supplementary L-carnitine demonstrated antioxidant effects in thermoneutral broilers [54], low-temperature stressed broilers, inducing ascites [49, 55] and high altitude broilers, inducing ascites [56], by virtue of its free radical scavenging properties [57]. To date, coenzyme Q and L-carnitine have not been tested in heat stressed poultry.

Enhancing ROS detoxifying capacity remains the primary target in heat stressed broilers, which can be achieved either by 1) scavenging ROS, 2) inhibiting enzymatic processes that lead to the formation of ROS or by chelating trace elements involved in ROS formation, or 3) by upregulating and protecting endogenous antioxidant defenses. In line with this, the possible role of the endogenous antioxidant GSH needs attention. Bioavailability of sufficient L-cysteine, the most limiting amino acid in GSH biosynthesis, guarantees appropriate synthesis of GSH, which in turn can be synthesized from methionine. Del Vesco et al. [58], Surai [59] showed that a diet in which methionine met the nutritional requirements resulted in lower OS upon acute HS in meat quail as compared to a diet deficient in methionine. Gursu et al. [41] and Sahin et al. [8] found that supplementing the diets of Japanese quail under conditions of cyclic chronic HS with 1 mg/kg folic acid (which plays a role in the methylation of homocysteine into methionine, amongst other functions) partially inverted the reductions in basal serum paraoxonase and arylesterase activities, vitamins A, B_12_, C, E, and folic acid, and reduced the increase of MDA in tissues. Attention is warranted in the choice of L-cysteine source (L-cysteine, L-cystine, or N-acetyl-L-cysteine) and the dietary level and source of methionine [45]. Many antioxidant enzymes have metals as a co-factor; therefore, dietary supply of these co-factors also needs thorough attention in situations of HS (e.g. [60]). The potential of the antioxidant vitamins A, E, and C are only briefly mentioned here; the reader is referred to the reviews of Khan et al. [61], Khan et al. [62], Lin et al. [5], and Mujahid [63] for an illustration of their established beneficial properties for heat stressed poultry. Some studies on these vitamins can also be found in Table 1; however, these are limited to studies in which these vitamins were applied as a comparison to the effect of phytochemicals. Vitamin C and vitamin E are the antioxidants of first choice to combat heat stress and are provided through the drinking water and feed, respectively, owing to their relatively short and long half-life in the animal’s body and different solubility properties.

**Table 1:** Effects of supplementary phytochemicals on oxidative status of poultry under cyclic chronic heat stress (CyCHS), constant chronic heat stress (CoCHS), and acute heat stress (AHS) (the birds were sampled at the end of HS, unless otherwise stated). Signicant effects on parameters of oxidative status are given for dietary treatments under HS conditions as compared to a heat stressed control (i.e. Cont. or T1). In case, both a non heat stressed (non supplemented) and heat stressed (non supplemented) control are included, data are used to confirm the presence of heat stress

Heat stress model	Poultry species and diets	Significant results	Reference
At d 10 of age for 32 d were exposed to: 1. Cont. at 22 °C 2. CyCHS at 34 °C for 8 h/d, (RH = 44 %) A 2×4 factorial design	Japanese quails; dietary treatments starting at d 10 of age 1. Cont.; 2. Cont. + genistein (GN) at 200 mg/kg; 3. Cont. + GN at 400 mg/kg; 4. Cont. + GN at 800 mg/kg	Serum, liver: MDA ↓ by GN *vs*. Cont. Serum: homocysteine ↓ by GN *vs*. Cont.; vitamins C, E, A ↑ by GN vs. Cont.	[43]
At wk 60 of age for 28 d all groups were exposed to CoCHS at 32 °C	Hy-Line laying hens; dietary treatments starting at wk 60 of age 1. Cont.; 2. Cont. + *Schisandra chinensis* (SC) at 10 g/kg; 3. Cont. + *Ligustrum lucidum* (LL) at 10 g/kg	Serum, liver, heart, egg yolk: MDA ↓ by SC and LL *vs*. Cont. Serum, kidney, liver: GR activity ↑ by SC and LL *vs*. Cont. Heart: GR activity ↑ by LL *vs*. Cont.	[64]
At d 10 of age for 32 d were exposed to: 1. Cont. at 22 °C, (RH = 57 %) 2. CyCHS at 34 °C for 8 h/d, (RH = 42 %) A 2×4 factorial design	Japanese quails; dietary treatments starting at d 10 of age 1. Cont.; 2. Cont. + lycopene at 50 mg/kg; 3. Cont. + lycopene at 100 mg/kg; 4. Cont. + lycopene at 200 mg/kg	Serum, liver, heart: MDA linear ↓ by lycopene *vs*. Cont. Serum: homocysteine linear ↓; vitamins C, E, A linear ↑; all by lycopene *vs*. Cont.	[69]
At d 0 of age for 49 d all groups were exposed to CoCHS at 38.6 ± 1.30 °C, (RH = 64 ± 6.0 %) On d 21 and 35 (RBS) and d 49 (other tissues) after starting HS birds were sampled	Cobb broilers; dietary treatments starting at d 0 of age 1. Cont., T1; 2. Cont. + vitamin E at 200 mg/kg, T2; 3. Cont. + dry powdered leaves of *Mentha longifolia* (DPLM) at 10 g/kg, T3; 4. Cont. + DPLM at 30 g/kg, T4; 5. Cont. + mix of *Emblica officinalis* fruit, vitamin E and electrolytes at 1 g/kg, T5	RBC: MDA ↓ by T2, T3, T4, T5 *vs.* T1 at d 21 and 35; GSH content ↑ by T3, T4, T5 *vs.* T1 at d 35; CAT, SOD, GR activity ↑ by T2, T3, T4, T5 *vs.* T1 at d 21 and 35 Heart, liver, brain cortex: MDA ↓ by T2, T3, T4, T5 *vs.* T1 at d 49; SOD, GR activity ↑ by T2, T3, T4, T5 *vs.* T1 at d 49	[67]
At d 3 of age for 39 d all groups were exposed to CoCHS at 32 ± 1 °C, (RH = 44 ± 6 %) On d 18 and 39 after starting HS birds were sampled	Male Arbor Acres broilers; dietary treatments starting at d 3 of age 1. Cont., T1; 2. Cont. + vitamin C at 200 mg/kg, T2; 3. Cont. + extract from dried fruits of *Forsythia suspensa* (FSE) at 100 mg/kg, T3	Serum: TAOC ↑ by T2, T3 *vs.* T1 at d 18 and 39; MDA ↓ by T2, T3 *vs.* T1 at d 18 and 39; SOD activity ↑ by T3 *vs.* T1 at d 18 Liver: MDA ↓ by T3 *vs.* T1 at d 39; SOD activity ↑ by T2, T3 *vs.* T1 at 18 and by T3 *vs.* T1 at d 39 Muscle: SOD activity ↑ by T3 *vs.* T1 at d 39; MDA ↓ by T2, T3 *vs.* T1 at d 39	[65]
At 1 kg BW for 20 d were exposed to: 1. Cont. at 30 °C, (RH = 65 %) 2. CyCHS at 40 ± 1 °C for 5 d out of 20 d for 4 h/d, (RH = 80 ± 5 %) 3. CyCHS at 40 ± 1 °C for 10 d out of 20 d for 4 h/d, (RH = 80 ± 5 %) A 3×2 factorial design	Male Gramapriya egg type domestic chickens (India); dietary treatments starting 10 d prior to CyCHS and during CyCHS or for 20 d (Cont. at 30 °C) 1. Cont.; 2. Cont. + Brahma Rasayana extract; Ayurvedic polyherbal preparation in which *Emblica officinalis* and *Terminalia chebula* are two major ingredients accounting ≥ 75 % *w/w* (BR) at 2 g/kg BW	RBC: CAT and SOD activities ↑ by BR *vs*. respective Cont. Serum, liver: MDA ↓ by BR *vs*. respective Cont. Liver: CAT, SOD, GSH-Px, GR activities ↑ by BR *vs*. respective Cont.	[44]
At d 10 of age for 32 d were exposed to: 1. Cont. at 22 °C 2. CyCHS at 34 °C for 8 h/d A 2×3 factorial design	Japanese quails; dietary treatments starting at d 10 of age 1. Cont.; 2. Cont. + epigallocatechin-3-gallate (EGCG) at 200 mg/kg; 3. Cont. + EGCG at 400 mg/kg	Serum, liver: MDA linear ↓ by EGCG *vs*. Cont. Serum: vitamins C, E, A ↑ by EGCG *vs*. Cont.	[70]
At d 0 of age for 41 d were exposed to: 1. Cont. at conventional temperature scheme (only Cont. diet) 2. CoCHS at 34 °C	Ross 308 broilers; dietary treatments starting at d 0 of age 1. Cont., T1; 2. Cont. + vitamin C at 250 mg/kg, T2; 3. Cont. + ethanol extract of propolis (EEP) at 0.5 g/kg, T3; 5. Cont. + EEP at 1 g/kg, T4; 5. Cont. + EEP at 3 g/kg, T5	Plasma: SOD activity ↓ by T2, T3, T4, T5 *vs*. T1; MDA ↓ by T2, T5 *vs*. T1; CAT activity ↓ by T5 *v.* T1; GSH-Px ↑ by T2, T4, T5 *vs*. T1 Liver: MDA ↓ by T5 *vs*. T1; CAT activity ↓ by T2, T5 *v.* T1; GSH-Px ↑ by T2, T3, T4, T5 *vs*. T1 Muscle: MDA ↓ by T4, T5 *vs*. T1; GSH ↓ by T2, T4, T5 *v.* T1 Kidney: CAT activity ↓ by T2, T5 *v.* T1; GSH ↓ by T4 *v.* T1; GSH-Px ↑ by T2, T3, T4, T5 *vs*. T1 Heart: CAT activity ↓ by T5 *v.* T1; GSH ↓ by T5 *v.* T1; GSH-Px ↑ by T2, T3, T4, T5 *vs*. T1	[38]
At d 18 of age for 27 d were exposed to: 1. Cont. at 26 ± 2 °C 2. CyCHS at 38 ± 2 °C for 6 h/d On d 1, 7, 14, 21 after starting HS birds were sampled	Male broilers; dietary treatments starting at d 18 of age 1. Cont., T1; 2. Cont. + polyphenols extracted from *Tamarindus indica* seed coat (PTSCE) at 100 mg/kg, T2; 3. Cont. + PTSCE at 200 mg/kg, T3; 4. Cont. + PTSCE at 300 mg/kg, T4; 5. Cont. + PTSCE at 400 mg/kg, T5; 6. Cont. + PTSCE at 500 mg/kg, T6	Serum: MDA ↓ by T5 *v.* T1 at d 1; MDA ↑ by T2, T3 *vs.* T1 at d 7 Serum (in average): MDA ↑ by T2 *vs.* T1	[68]
At d 0 for 42 d all groups were exposed to CoCHS at 32.86 ± 0.68 °C On d 21 and 42 after starting HS birds were sampled	Cobb broilers; dietary treatments starting at d 0 of age 1. Cont., T1; 2. Cont. + polyherbal mix Stresroak (fruits and leaves from different herbs, containing vitamin C and flavonoids as major active principles) at 1 g/kg, T2; 3. Cont. + vitamin C at 100 mg/kg, T3	Serum: SOD activity ↑ by T2, T3 *vs*. T1 at d 21; SOD activity ↑ by T2 *vs*. T1 at d 42; GR activity ↑ by T2, T3 *vs*. T1 at d 21 and 42	[66]
At d 35 for 12 wk were exposed to: 1. Cont. at 22 °C 2. CyCHS at 34 °C for 8 h/d A 2×3 factorial design	Female Japanese quails; dietary treatments starting at d 35 of age 1. Cont.; 2. Cont. + epigallocatechin-3-gallate (EGCG) at 200 mg/kg; 3. Cont. + EGCG at 400 mg/kg	Liver: MDA, NF-kB linear ↓ by EGCG *vs*. Cont.; CAT, SOD, GSH-Px activity, Nrf2 inear ↑ by EGCG *vs*. Cont	[29]
At d 35 for 12 wk were exposed to: 1. Cont. at 22 °C 2. CyCHS at 34 °C for 8 h/d A 2×3 factorial design	Female Japanese quails; dietary treatments starting at d 35 of age 1. Cont.; 2. Cont. + tomato powder (TP) at 25 g/kg; 3. Cont. + TP at 50 g/kg	Liver: MDA, NF-kB linear ↓ by TP *vs*. Cont.; CAT, SOD, GSH-Px activity, Nrf2 inear ↑ by TP *vs*. Cont	[71]
At d 35 for 12 wk were exposed to: 1. Cont. at 22 °C 2. CyCHS at 34 °C for 8 h/d A 2×3 factorial design	Female Japanese quails; dietary treatments starting at d 35 of age 1. Cont.; 2. Cont. + *Berberis vulgaris* root extract (BVE) at 200 mg/kg; 3. Cont. + BVE at 400 mg/kg	Liver: MDA, HSP70, NF-kB linear ↓ by EGCG *vs*. Cont.; CAT, SOD, GSH-Px activity, HO-1, Nrf2 linear ↑ by EGCG *vs*. Cont	[72]
At d19 for 5 d were exposed to: 1. Cont. at 24 °C 2. CoCHS at 34 °C (RH = 55 %) A 2×4 factorial design	Ross 308 broilers; dietary treatments starting at d 0 of age 1. Cont., T1; 2. Cont. + cashew nut shell liquid (CNSL, 75 % anacardic acids) at 2 mg/kg, T2; 3. Cont. + grape seed extract (GSE, 40 % proanthocyanidins) at 40 mg/kg, T3; 4. Cont. + electrolysed reduced water (ERW, pH 8.1 to 10.1; Eh -160 to -607 mV), T4	Pectorial superficialis muscle: H_2_O_2_ ↓ by T3 vs. T1	[47]
At d 28 for 10 d all groups were exposed to CyCHS at 34 °C for 5 h/d followed by 22 °C, (RH = 50 %)	Ross 308 broilers; dietary treatments starting at d 25 of age 1. Cont., T1; 2. Cont. + *Curcuma xanthorrhiza* essential oil (CXEO) at 200 mg/kg, T2; 3. Cont. + CXEO at 400 mg/kg, T3; 4. Cont. + lemon peel extract (LPE) at 200 mg/kg, T4; 5. Cont. + LPE at 400 mg/kg, T5; 2. Cont. + orange peel extract (OPE) at 200 mg/kg, T6; 3. Cont. + OPE at 400 mg/kg, T7	RBC: GSH-Px activity ↑ by T2, T3, T7 *vs.* T1; SOD activity ↑ in T3 *vs.* T1	[78]
At d 42 for 15 d were exposed to: 1. Cont. at 24 ± 2 °C (only Cont.diet) 2. CyCHS at 37 ± 2 °C for 8 h/d followed by 24 ± 2 °C	Female Xuefeng black-boned chickens; dietary treatments starting at d 42 of age 1. Cont.; 2. Cont. + 200 mg/kg resveratrol; 3. Cont. + 400 mg/kg resveratrol; 4. Cont. + 600 mg/kg resveratrol	Serum: MDA linear ↓; GSH linear ↑; GSH-Px, SOD and CAT activity quadratic ↑; all resveratrol *vs*. Cont. Bursa of Fabricius: HSP27 mRNA levels linear ↓; HSP70 and HSP90 mRNA levels quadratic ↓; all resveratrol *vs*. Cont. Thymus: HSP27and HSP90 mRNA levels linear ↑; HSP70 mRNA levels linear ↓; all resveratrol *vs*. Cont. Spleen: HSP27, HSP70 and HSP90 mRNA levels quadratic ↓; all resveratrol *vs*. Cont.	[74]
At d 14 for 26 d all groups were exposed to CyCHS at 32 ± 2 °C for 8 h/d followed by 19-24 °C On d 21 after starting HS birds were sampled	Cobb 500 male broilers; dietary treatments starting at d 0 of age 1. Cont., T1; 2. Cont. + 100 mg/kg vitamin E, T2; 3. Cont. + 7.5 mg/kg ginger root powder (GRP), T3; 4. Cont. + 15 mg/kg GRP, T4; 5. Cont. + 75 mg/kg ginger essential oil (GEO), T5; 6. Cont. + 150 mg/kg GEO, T6	RBC: SOD activity ↓ by T5 + T6 *vs*. T1 Serum: Total antioxidant capacity ↑ by T2, T3, T4, T5, T6 *vs.* T1; MDA ↓ by T2, T3, T4, T5, T6 *vs*. T1 Liver: SOD activity ↑ by T5 + T6 *vs*. T1; MDA ↓ by T3, T4, T5, T6 *vs*. T1	[75]
At d 28 for 14 d all groups were exposed to CyCHS at 34 °C for 5 h/d followed by 22 °C, (RH = 50-60 %) On d 3 and 14 after starting HS birds were sampled	Ross 308 broilers; dietary treatments starting at d 25 of age 1. Cont., T1; 2. Cont. + *Curcuma xanthorrhiza* essential oil (CXEO) at 200 mg/kg, T2; 3. Cont. + CXEO at 400 mg/kg, T3; 4. Cont. + *Oreganum compactum* essential oil (OCEO) at 200 mg/kg, T4; 5. Cont. + OCEO at 400 mg/kg, T5	Plasma: MDA ↓ by T3 *vs.* T1 at d3; ↓ by T2, T3, T4 *vs.* T1 at d 14 RBC: GSH content ↑ by T2, T3, T4, T5 *vs.* T1 at d 14 Liver: CAT activity ↑ by T2, T5 *vs.* T1 at d 14 d; GSH-Px activity ↑ by T2 *vs.* T1 at d 14; SOD activity ↑ by T3 *vs.* T1 at d 3; HSP70 mRNA levels ↓ by T5 *vs.* T1 at d 3 Kidney: SOD activity ↑ by T3, T5 *vs.* T1; SOD mRNA levels ↑ by T5 *vs.* T1; HSP70 mRNA levels ↓ by T5 *vs.* T1 at d 3 Heart: CAT activity ↑ by T3, T5 *vs.* T1; GSH-Px activity ↑ by T2, T3, T5 *vs.* T1; SOD activity ↑ by T2, T3 *vs.* T1; CAT mRNA levels ↑ by T3, T5 *vs.* T1; SOD mRNA levels ↑ by T3 *vs.* T1 all at d 3; HSP70 mRNA levels ↓ by T3 *vs.* T1 at d 14	[79]

RH, relative humidity; MDA, malondialdehyde; GN, genistein; SC, *Schisandra chinensis*; LL, *Ligustrum lucidum*; GR, glutathione reductase; DPLM, dry powdered leaves of mint; RBC, red blood cell; GSH, glutathione; CAT, catalase; SOD, superoxide dismutase; FSE, *Forsythia suspense* extract; TAOC, total antioxidant capacity; BR, B*rahma rassayana* (made by the mixing extracts from plants); EGCG, epigallocatechin-3-gallate; H_2_O_2_, hydrogen peroxide; avUCP, avian uncoupling proteins; GSH-Px, glutathione peroxidase; EEP, ethanol extract of propolis; PTSCE, polyphenols extracted from *Tamarindus indica* seed coat; Nrf2, nuclear factor erythroid 2–related factor 2; GSSG, glutathione disulfide; BVE, *Berberis vulgaris* root extract; HO-1, haeme oxygenase-1; GST, glutathione-S-transferase; CNSK, cashew nut shell liquid; GSE, grape seed extract; ERW, electrolysed reduced water; CXEO, *Curcuma xanthorrhiza* essential oil; OCEO, *Oreganum compactum* essential oil; HSP, heat shock protein; LPE, lemon peel extract; OPE, orange peel extract; GRP, ginger root powder; GEO, ginger essential oil

### Antioxidant and favorable properties of phytochemicals

A literature survey was performed to collect data on the effects of feeding supplementary phytochemicals with demonstrated antioxidant properties to poultry under HS on oxidative status parameters (Table 1). The effects on other physiological responses and bird performance are not considered here. Phytochemicals are here defined as any product derived from plants containing secondary plant metabolites as active principles (e.g. dried plant material, extract, essential oil, pure isolated compound). All experiments were conducted in chronic HS models except one case. Surprisingly few studies have been reported in the literature, most of which showed a positive outcome. Two herbs used in traditional Chinese medicine were shown to reduce MDA in the tissues and egg yolk of heat stressed laying hens [64]. The seeds of *Schisandra chinensis* contain lignans, while the seeds of *Ligustrum lucidum* contain oleanolic acid (a triterpenoid) amongst other principles. An extract from the dried fruits of *Forsythia suspensa* (another herb used in traditional Chinese medicine and containing the lignan pinoresinol) at 100 mg/kg showed a greater capacity than 200 mg/kg vitamin C in alleviating oxidative damage upon heat stress [65]. Further, Ramnath et al. [44] and Sujatha et al. [66] demonstrated that Brahma Rasayana extract and Stressroak, which are Ayurvedic polyherbal preparations, could relieve heat stress as compared to a control diet. Stressroak contains vitamin C and flavonoids as the active principles. The major Brahma Rasayana herbs are gooseberry (*Emblica officinalis*) and Indian gall nut (*Terminalia chebula*). In the study of Ramnath et al. [44], the preparation was fed starting 10 d prior to HS. Maini et al. [67] also tested a product containing fruits of *E. officinalis*, however, this time in a mixture with vitamin E and electrolytes. Its positive effects on red blood cells, heart, liver, and the brain cortex of constant chronic heat stressed broilers can thus not be ascribed solely to the presence of the herb. The same authors found also that dry powdered leaves of *Mentha longifolia* at 10, 20, and 30 g/kg were beneficial. Seven et al. [38] supplemented diets of constant chronic heat stressed broilers with increasing amounts of an ethanol extract of propolis. In particular the higher dose (3 g/kg) was able to ameliorate the oxidative status in different tissues similar to 250 mg/kg vitamin C. They identified 22 components in the extract; farnesol (a sesquiterpenoid) being the most abundant, followed by the flavone chrisin, 1-propen-1thiol, and 1-cyclohexene-1-methanol. Adding polyphenols extracted from *Tamarindus indica* seed coats did not provoke alleviation of oxidative damage upon increased temperatures [68]. Sahin and co-workers tested the efficacy of genistein (an isoflavone flavonoid; [43]), lycopene (a carotene with no vitamin A activity; [69]), epigallocatechin-3-gallate (a flavanol flavonoid; [29, 70]), tomato powder (containing 0.80 mg lycopene, 0.13 mg β-carotene, 0.07 mg α-tocopherol, 1.73 mg vitamin C per g of powder; [71]), and *Berberis vulgaris* root extract (chemical composition given in [72]), in cyclic heat stress models with Japanese quails. They found beneficial effects, and for some phytochemicals they gave evidence that this was achieved by activating the host defense system at the cellular level, substantiated by up-regulation of the transcription factor Nrf2 and down-regulation of NF-κB [29, 71, 72]. Sahin et al. [71] demonstrated high and significant correlations between feed intake and egg production on the one hand and Nrf2 (positive) and NF-κB (negative) on the other, irrespective of epigallocatechin-3-gallate supplementation addressing their role in mediating oxidative stress responses. Sahin et al. [73] reviewed possible molecular targets of dietary phytochemicals for the alleviation of heat stress in poultry. *In concreto*, the cell signaling modulating effects of epigallocatechin-3-gallate, lycopene, resveratrol, and curcumin are discussed. Sahin et al. [69] demonstrated that by supplementing lycopene, its serum concentration increased dose-dependently. The positive effects of resveratrol (a stillbenoid) were shown by Liu et al. [74] in a dose–response study. Resveratrol attenuated the heat stress induced over expression of HSP27, HSP70, and HSP90 mRNA in the bursa of Fabricius and spleen, and increased the low expression of HSP27 and HSP90 mRNA in the thymus upon heat stress. It also showed positive effects on oxidative status parameters in serum. The heat-shock response, through activation of heat-shock transcription factors and the elevated expression of inducible HSP and molecular chaperones, protects the cell against the accumulation of non-native proteins. HSPs interact with those proteins to hold them together properly and retain their functionality, hence exhibiting a major contribution to cell integrity under these stress conditions. Proper expression of HSP is fundamental to the protective heat stress response. Azad et al. [47] showed clearly that electrolyzed reduced water (not a phytochemical, but mentioned here for comparison) could alleviate the negative effects of HS on feed consumption and body weight gain, while it had no significant effects on MDA and H_2_O_2_ formation in muscles. Electrolyzed reduced water was provided to the chickens as a source of “active hydrogen” which is believed to act as a ROS scavenger without producing oxidized molecules after reduction. Conversely, grape seed extract [rich in proanthocyanidins (oligomers of the flavanols catechin and epicatechin) vitamin E, and other flavonoids] significantly reduced H_2_O_2_ production in muscles, whereas no effect was seen on performance. Habibi et al. [75] worked with ginger root powder (7.5 and 15 mg/kg) and a hydro distillate thereof (essential oil; 75 and 150 mg/kg); the latter having 12 constituents identified, with zingiberene, β-sesquiphellandrene, sabinene, ar-curcumene, and β-bisabolene as major components; all being sesquiterpenes except sabinine which is a monoterpene. All treatments, including the positive control vitamin E (100 mg/kg) improved serum oxidative status, but only the ginger oil increased SOD activity in the liver and both ginger root and oil reduced liver MDA as compared to controls. Recently, our studies have shown that phenolic compounds produce beneficial effects on poultry owing to their antioxidant, anti-inflammatory, and antimicrobial activities [76, 77]. Moreover, in other work from our group [78, 79], we have shown that *Curcuma xanthorrhiza* essential oil, *Oreganum compactum* essential oil, and orange peel extract are able to improve the oxidative status of broiler chickens under cyclic chronic HS (28-day-old Ross 308 broilers were exposed to 34 °C for 5 h/d followed by 22 °C for 14 d). *Curcuma xanthorrhiza* essential oil is characterized by ar-curcumene (11.4 %), β-curcumene (8.5 %; sesquiterpenes), and xanthorrizhol (28 %, a sesquiterpenoid) and in both studies showed significant improvements in the oxidative status of various tissues, mainly at the higher inclusion of 400 mg/kg. *Oreganum compactum* essential oil, containing the monoterpenoids carvacrol (44.9 %) and thymol (16.4 %) showed inferior improvements almost exclusively at the higher dose (400 mg/kg). The significant effects of orange peel extract were limited to increasing erythrocyte GSH-Px activity for the 400 mg/kg treatment, likely because of the low content of active principles in the extract.

It appears to be difficult to identify a consistent pattern of functional antioxidant phytochemicals. Nevertheless, several studies found positive results with terpenes/terpenoids, and in particular with sesquiterpenes/sesquiterpenoids (ethanol extract of propolis, *Curcuma xanthorrhiza* essential oil, and ginger root oil). Lignans in traditional Chinese medicine herbs and lycopene have also showed positive results. Nonetheless, in the majority of the studies the beneficial principles were various types of flavonoids and related compounds (proanthocyanidins and resveratrol), either in combination or not with vitamin C or E. Surai [79] questioned the *in vivo* direct antioxidant effects of flavonoids, basically because these compounds are poorly absorbed in the gut, rapidly metabolized and excreted resulting in physiologically low concentrations in target tissues (e.g. mostly lower than 1 μmol/L in the plasma of healthy subjects). In none of the studies using flavonoids from Table 1 were the tissue concentrations of the active principle measured. In most studies shown in Table 1 it was reported that these phytochemicals were beneficial to heat stressed poultry but were less or ineffective in non-heat-stressed poultry (e.g. [43, 70, 71]; this supports the contention that antioxidant phytochemicals might have potential in challenging conditions. None of the beneficial effects of these phytochemicals were confirmed in a second study, except for those associated with epigallocathechin-3-gallate and *Curcuma xanthorrhiza* essential oil, which were tested twice by the same research groups ([29, 70] and [78, 79]; respectively). Therefore, we suggest that more studies are needed in this subject; in particular, well-designed dose–response studies (dose effect is poorly addressed in the above studies) including non-heat stressed control treatments that investigate the mode of action are warranted.

## Conclusions

Current evidence strongly suggests that HS induces oxidative stress in poultry. The current review focused on mitochondrial dysfunction upon heat stress. It is suggested that mitigation of mitochondrial dysfunction can be approached though various nutritional strategies: 1) reduction of the membrane potential (e.g. mild uncoupling), 2) improvement of ETC. efficiency, or 3) enforcement of the host’s capacity to detoxify ROS. Currently, only evidence with regard to the latter strategy is available. Several studies found positive results with terpenes/terpenoids, and in particular with sesquiterpenes/sesquiterpenoids (ethanol extract of propolis, *Curcuma xanthorrhiza* essential oil, and ginger root oil). Lignans in traditional Chinese medicinal herbs and lycopene showed positive results. Nonetheless, in the majority of studies, the beneficial principles were various types of flavonoids and related compounds (proanthocyanidins and resveratrol), either in, or not in combination with vitamin C or E. Epigallocatechin-3-gallate and lycopene were found to interact with cell signaling pathways. It was repeatedly reported that these phytochemicals were beneficial to heat stressed poultry but were less or not effective in non-heat-stressed control birds. This supports the contention that antioxidant phytochemicals might have potential in challenging conditions.

## Abbreviations

2-Cys PRX, 2-Cys peroxiredoxin; 3HADH, 3-hydroxyacyl CoA dehydrogenase; ANT, adenine nucleotide translocator; AP-1, activator protein 1; ARE, antioxidant response element; avUCP, avian uncoupling protein; CCO, cytochrome c oxidase; CNSL, cashew nut shell liquid; CS, citrate synthase; ETC., electron transport chain; FADH2, flavin adenine dinucleotide; GSH, reduced glutathione; GSH-Px, glutathione peroxidase; GSSG, glutathione disulfide; HS, heat stress; HSP, heat-shock proteins; MDA, malondialdehyde; NCCR, nicotinamide adenine dinucleotide cytochrome c reductase; NF-κB, nuclear factor kappa-light-chain-enhancer of activated B; Nrf2, nuclear factor (erythroid-derived 2)-like 2; OS, oxidative stress; ROS, reactive oxygen species; RS, reactive species; SCCR, succinate cytochrome c reductase; SOD, superoxide dismutase; TCA, tricarboxylic acid cycle; UCP, Uncoupling proteins

## Additional files

Additional file 1:
**Table S1.** Effects of acute heat stress (HS) on oxidative status of poultry (the birds were sampled at the end of HS, unless otherwise stated). Cont. refers to non heat stressed birds. (DOCX 41 kb) Additional file 2:
**Table S2.** Effects of cyclic chronic heat stress (CyCHS) and constant chronic heat stress (CoCHS) on oxidative status of poultry (the birds were sampled at the end of HS, unless otherwise stated). Cont. refers to non heat stressed birds. (DOCX 44 kb)
