# Supplementary material for: Association between heat stress and oxidative stress in poultry; mitochondrial dysfunction and dietary interventions with phytochemicals
Source: J Anim Sci Biotechnol. 2016 Jun 28;7:37. doi: 10.1186/s40104-016-0097-5 (PMC4924307; doi:10.1186/s40104-016-0097-5)
Supplement: Additional file 1: — Table S1. Effects of acute heat stress (HS) on oxidative status of poultry (the birds were sampled at the end of HS, unless otherwise stated). Cont. refers to non heat stressed birds. (DOCX 41 kb) [file 40104_2016_97_MOESM1_ESM.docx]

| **Supplementary Table S1** *Effects of acute heat stress (HS) on oxidative status of poultry (the birds were sampled at the end of HS, unless otherwise stated). Cont. refers to non heat stressed birds* | | |
| --- | --- | --- |
| Poultry species and Treatments | Significant results (HS *v.* Cont.) | Reference |
|  |  |  |
| Cobb and Ross broilers  At d 35 and 36 for 3 h were exposed to:  1. Cont. at 20±2°C  2. HS at 38±1°C, (RH>50%) | In both breeds:  **Whole blood:** CAT, GSH-Px, and SOD activities and MDA ↑ | Altan *et al*. 2003 |
|  |  |  |
| Cobb meat and laying-type chickens  At d 16 for 18 h were exposed to:  1. Cont. at 25°C  2. HS at 34°C | **Pectoralis superficialis muscle of meat type chickens:** ROS ↑ | Mujahid *et al*. 2005 |
|  |  |  |
| Cobb broilers  At d 21 and d 35 for 18 h were exposed to:  1. Cont. at 25°C  2. HS at 34°C | **Pectoralis superficialis mitochondria:** at d 35: ROS ↑  **Pectoralis superficialis muscle:** at d 21: avUCP mRNA levels ↓  **Pectoralis superficialis mitochondria:** at d 21: avUCP mRNA levels ↓ | Mujahid *et al*. 2006 |
|  |  |  |
| Cobb broilers  At d 35 for 6 h were exposed to:  1. Cont. at 21°C, (RH=45%)  2. HS at 32°C, (RH=40%)  At 3 and 6 h after starting HS were sampled. | **Plasma:** MDA ↑ (after 3 and 6 h); FRAP ↓ (after 3 h)  **Liver:** MDA ↑ (after 3 and 6 h); FRAP ↑ (after 6 h)  **Heart:** SOD activity ↑ (after 6 h) | Lin *et al*. 2006 |
|  |  |  |
| Cobb broilers  At d 32 for 18 h were exposed to:  1. Cont. at 25°C  2. HS at 34°C, (RH=55±5%) | **Plasma, Pectoralis superficialis muscle:** MDA ↑  **Pectoralis superficialis muscle:** protein carbonyl ↑ | Mujahid *et al*. 2007c |
|  |  |  |
| Julia-Leghorn laying type male chickens  At d 16 and d 87 for 18 h were exposed to:  1. Cont. at 25°C and 21°C  2. Chicks (d 16): HS at 34°C  3. Cockerels (d 87): HS at 34°C | **Pectoralis superficialis mitochondria:** ROS ↑ in cockerels  **Pectoralis superficialis muscle:** avUCP mRNA levels ↓ in cockerels | Mujahid *et al*. 2007a |
|  |  |  |
| Cobb broilers  At d 16 for 18 h were exposed to:  1. Cont. at 25°C  2. HS at 34°C, (RH=55±5%)  At 6, 12, 18 h after starting HS were sampled. | **Pectoralis superficialis mitochondria:** ROS ↑ (after all times); avUCP mRNA levels ↓ (after all times); 3HADH and CS activities ↑ (after 6 h)  **Sarcoplasma:** avUCP protein levels ↓ (after all times) | Mujahid *et al*. 2007b |
|  |  |  |
| Arbor Acres broilers  At d 30 for 5 h were exposed to:  1. Cont. at 25±1°C  2. HS at 40±1°C, (RH=53-60%)  At 1, 2, 3, 5 h after starting HS were sampled. | **Pectoralis major muscle:** MDA ↑ (after 2, 3, 5 h)  **Sarcoplasma:** protein carbonyl ↑ (after 2, 3, 5 h)  **Myofibril:** protein carbonyl ↑ (after all times) | Wang *et al*. 2009 |
|  |  |  |
| Cobb broilers  At d 16 for 12 h were exposed to:  1. Cont. at 25°C  2. HS at 34°C, (RH=55±5%) | **Pectoralis superficialis muscle:** avUCP mRNA levels ↓  **Pectoralis superficialis mitochondria:** avUCP protein band integrity ↓  **Pectoralis superficialis muscle and mitochondria:** MDA ↑ | Mujahid *et al*. 2009 |
|  |  |  |
| Arbor Acres broilers  At d 42 for 3 h were exposed to:  1. Cont. at 25°C  2. HS at 32°C  3. HS at 35°C  4. HS at 38°C | All 3 treatments *v.* Cont.:  **Serum, liver:** SOD, GSH-Px, CAT activities, and MDA ↑  **Liver:** protein carbonyl ↑; NCCR and CCO activities ↓ | Tan *et al*. 2010 |
|  |  |  |
| Arbor Acres male broilers  At d 49 for 3 h were exposed to:  1. Cont. at 25°C  2. HS at 35°C, (RH=70±5%)  At 0, 1, 2, 4, 8, 12 h after the end of HS were sampled. | **Serum:** GSH-Px activity and MDA levels ↑ (all times); CAT activity ↑ (at 0, 1, 2 h of HS)  **Liver:** ROS, SOD activity, and MDA ↑, NCCR and CCO activities ↓ (all times); CAT activity ↑ (at 0, 1, 2, 4 h of HS); GSH-Px activity ↑ (after 0, 1, 2, 4, 8, 12 h of HS) | Yang *et al*. 2010 |
|  |  |  |
| Cobb broilers  At d 21 for 12 h were exposed to:  1. Cont. at 24°C  2. HS at 34°C, (RH=55±5%) | **Pectoralis superficialis muscle:** MDA, protein carbonyl, H_2_O_2_ production ↑ | Kikusato and Toyomizu 2013 |
| Japanese quail  At d 30 for 24 h were exposed to:   1. Cont. at 25°C 2. HS at 34±0.6°C (RH=60±0.4%) | **Liver:** ROS and GSH-Px mRNA ↑; avUCP mRNA levels ↓ | Del Vesco and Gasparino 2013 |
| Meat quail  At d 41 for 24 h were exposed to:   1. Cont. at 25°C 2. HS at 38°C | **Liver:** ROS, GSH-Px activity, CAT activity ↑  **Plasma:** uric acid ↑ | Del Vesco *et al*. 2014 |
| RH, relative humidity; CAT, catalase; GSH-Px, glutathione peroxidase; SOD, superoxide dismutase; MDA, malondialdehyde; ROS, reactive oxygen species; avUCP, avian uncoupling proteins; FRAP, ferric reducing ability of plasma; 3HADH, 3-hydroxylacyl CoA dehydrogenase; CS, citrate synthase; NCCR, nicotinamide adenine dinucleotide (NADPH) cytochrome c reductase; CCO, cytochrome C oxidase; TBARS, thiobarbituric acid reactive substances | | |
