# Supplementary material for: Association between heat stress and oxidative stress in poultry; mitochondrial dysfunction and dietary interventions with phytochemicals
Source: J Anim Sci Biotechnol. 2016 Jun 28;7:37. doi: 10.1186/s40104-016-0097-5 (PMC4924307; doi:10.1186/s40104-016-0097-5)
Supplement: Additional file 2: — Table S2. Effects of cyclic chronic heat stress (CyCHS) and constant chronic heat stress (CoCHS) on oxidative status of poultry (the birds were sampled at the end of HS, unless otherwise stated). Cont. refers to non heat stressed birds. (DOCX 44 kb) [file 40104_2016_97_MOESM2_ESM.docx]

| **Supplementary Table S2** *Effects of cyclic chronic heat stress (CyCHS) and constant chronic heat stress (CoCHS) on oxidative status of poultry (the birds were sampled at the end of HS, unless otherwise stated). Cont. refers to non heat stressed birds* | | |
| --- | --- | --- |
| Poultry species and Treatment | Significant results (HS *v.* Cont.) | Reference |
|  |  |  |
| Japanese quails  At d 10 for 32 d were exposed to:  1. Cont. at 22°C  2. CyCHS at 34°C for 8 h/d | **Serum, liver, heart, kidney:** MDA ↑  **Serum:** homocysteine ↑; vitamins C, E, A, Folic acid, B_12_ ↓; retention of Zn, Cu, Fe, Cr ↓ | Sahin *et al*. 2003 |
|  |  |  |
| Laying Japanese quails  At 13 wk for 3 wk were exposed to:  1. Cont. at 22°C  2. CoCHS at 34°C, (RH=42%) | **Serum, liver:** MDA ↑;  **Serum:** vitamins C, E, Zn ↓ | Sahin and Kucuk, 2003 |
|  |  |  |
| Japanese quails  At d 10 for 32 d were exposed to:  1. Cont. at 22°C  2. CyCHS at 34°C for 8 h/d, (RH=44%) | **Serum, liver:** MDA ↑  **Serum:** homocysteine ↑; vitamins C, E, A ↓ | Onderci *et al*. 2004 |
|  |  |  |
| Japanese quails  At d 10 for 32 d were exposed to:  1. Cont. at 22°C  2. CyCHS at 34°C for 8 h/d | **Serum, liver, heart, kidney:** MDA ↑  **Serum:** homocysteine ↑; vitamins C, E, A, Fe, Zn, Cu, Cr, basal paraoxonase, NaCl-stimulated paraoxonase, arylesterase ↓ | Sahin *et al*. 2004b |
|  |  |  |
| Japanese quails  At d 10 for 32 d were exposed to:  1. Cont. at 22°C  2. CyCHS at 34°C for 8 h/d | **Serum, liver:** MDA ↑; vitamins C, E, and A ↓  Excretion of Ash, Ca, P, Mg, Zn, Fe, Cr ↑ | Sahin *et al*. 2004a |
|  |  |  |
| Japanese quails  At d 10 for 30 d were exposed to:  1. Cont. at 22°C  2. CyCHS at 34°C for 8 h/d | **Serum:** basal paraoxonase, NaCl-stimulated paraoxonase, arylesterase, Albumin ↓ | Gursu *et al*. 2004 |
|  |  |  |
| Japanese quails  At d 10 for 32 d were exposed to:  1. Cont. at 22°C  2. CyCHS at 34°C for 8 h/d | **Serum, liver:** MDA ↑  **Serum:** vitamins E, C, A ↓ | Sahin *et al*. 2005b |
|  |  |  |
| Japanese quails  At d 10 for 32 d were exposed to:  1. Cont. at 22°C  2. CyCHS at 34°C for 8 h/d | **Serum, liver, thigh meat:** MDA ↑ | Sahin *et al*. 2005a |
|  |  |  |
| Japanese quails  At d 10 for 32 d were exposed to:  1. Cont. at 22°C, (RH=57%)  2. CyCHS at 34°C for 8 h/d (RH=42%) | **Serum, liver, heart:** MDA ↑  **Serum:** homocysteine ↑; vitamins C, E, A ↓ | Sahin *et al*. 2006b |
|  |  |  |
| Japanese quails  At d 10 for 32 d were exposed to:  1. Cont. at 22°C  2. CyCHS at 34°C for 8 h/d | **Serum, liver:** MDA ↑  **Serum:** Zn, vitamins C, E ↓ | Sahin *et al*. 2006a |
|  |  |  |
| Male Gramapriya egg type domestic chickens (India)  At 1 kg BW for 20 d were exposed to:  1. Cont. at 30°C, (RH=65%)  2. CyCHS at 40±1°C for 5 d out of 20 d for 4 h/d, (RH=80±5%)  3. CyCHS at 40±1°C for 10 d out of 20 d for 4 h/d, (RH=80±5%) | **Serum, liver:** MDA ↑ at d 5 and 10  **Serum:** CAT activity ↑ at d 5 and 10; SOD activity ↓ at d 5 and 10  **Liver:** GSH content ↑ at d 5 and 10; CAT, SOD activities ↑ at d 5 and 10; GSH-Px activity ↑ only at d 5; GR activity ↓ at d 5 and 10 | Ramnath *et al*. 2008 |
|  |  |  |
| Arbor Acres broilers  At wk 4 for 3 wk were exposed to:  1. Cont. at 22°C  2. CyCHS 28°C to 34°C; time not mentioned | **Liver mitochondria:** H_2_O_2_ production ↑  **Liver, breast:** MDA ↑ | Feng *et al*. 2008 |
|  |  |  |
| Japanese quails  At d 10 for 32 d were exposed to:  1. Cont. at 22°C  2. CyCHS at 34°C for 8 h/d | **Serum, liver:** MDA ↑  **Serum:** vitamins C, E, A ↓ | Tuzcu *et al*. 2008 |
|  |  |  |
| Japanese quails  At d 55 for 90 d were exposed to:  1. Cont. at 22°C  2. CyCHS at 34°C for 8 h/d | **Serum, liver, egg yolk:** MDA ↑  **Serum, egg white, egg yolk:** Se ↓ | Sahin *et al*. 2008 |
|  |  |  |
| ISA JV 15 broilers  At d 28 for 10 d were exposed to:  1. Cont. at 22°C  2. CoCHS at 32°C | **Leg muscle:** UCP rRNA levels ↑ | Dridi *et al*. 2008 |
|  |  |  |
| Broilers  At d 28 for 21 d were exposed to:  1. Cont. at 26±2°C  1. CoCHS at 38±2°C for 1, 4, 7, 11, 21 d | **Serum:** MDA ↑ at d 1, 4, 7, 11  **RBC:** GSH-Px activity ↑ at d 1, 4, 7, 11 | Pamok *et al*. 2009 |
|  |  |  |
| Ross broilers  At d 0 for 41 d were exposed to:  1. Cont. at 21°C  2. CoCHS at 34°C | **Plasma:** MDA, SOD and CAT activity ↑; GSH-Px activity ↓  **Liver:** MDA, GSH, CAT activity ↑; GSH-Px activity ↓  **Muscle:** MDA, GSH ↑  **Kidney:** GSH, CAT activity ↑; GSH-Px activity ↓  **Heart:** GSH, CAT activity ↑; GSH-Px activity ↓ | Seven *et al*. 2009 |
|  |  |  |
| Ross broilers  At d 14 for 14 d were exposed to:  1. Cont. at 24°C  2. CyCHS ranging from 32°C to 24°C to 32°C (32°C for 8 h/d)  3. CoCHT at 32°C  4. CoCHS at 34°C | **Plasma:** uric acid ↑ (in constant 34)  **Pectorial superficialis muscle:** 3HADH activity ↓ (in cyclic and constant 34); CS activity ↓, Cu/Zn SOD activity ↑ (in constant 34); MDA ↑ (in both constant); avUCP rRNA levels ↑ (in all groups) | Azad *et al*. 2010b |
|  |  |  |
| Ross broilers  At d 19 for 14 d were exposed to:  1. Cont. at 24°C  1. CoCHS at 34°C for 1, 3, 5, 9, 14 d and were sampled after each time, (RH=55±5%) | **Pectorial superficialis mitochondria:** H_2_O_2_ production ↑ at d 5 and 9; membrane potential in state 4 ↑ at d 5; oxygen consumption in state 3 ↑ at d 5; 3HADH activity , FCCP-stimulated oxygen consumption ↑ at d 9; CS activity ↓ at d 14; SOD, CAT activities ↑ at d 14 | Azad *et al*. 2010a |
|  |  |  |
| Female Japanese quails  At d 35 for 12 wk were exposed to:  1. Cont. at 22°C  2. CyCHS at 34°C for 8 h/d | **Liver:** NF-κB expression, MDA ↑; SOD, GSH-Px, CAT activities, Nrf2 expression ↓ | Sahin *et al*. 2010 |
|  |  |  |
| Japanese quails  At d 21 for 21 d were exposed to:  1. Cont. at 24°C  2. CyCHS at 34°C for 9 h/d | **Pectorial superficialis muscle:** SOD activity, MDA ↑; GSH content, CAT activity ↓ | Halici *et al*. 2012 |
|  |  |  |
| Ross male broilers  At d 14 for 4 wk were exposed to:  1. Cont. decreased from 25.5°C to 18°C  2. CoCHS at 32°C  On wk 2, 4 after starting HS were sampled. | **Plasma:** uric acid ↑ after 2 and 4 week  **Liver:** total GSH, GSSG ↑; reduced GSH: total GSH, and reduced GSH: GSSG ↓ after 4 week | Willemsen *et al*. 2011 |
|  |  |  |
| Hubbard broilers  At d 22 for 20 d were exposed to:  1. Cont. at 26.7°C, (RH=65±5%)  2. CyCHS at 35±1.1°C for 8 h/d, (RH=75±5%) | **Serum:** total oxidants (µm H_2_O_2_ equivalent) and total antioxidants (m*M* equivalent of vitamin C), ceruloplasmin ↑; paraoxonase, arylesterase ↓ | Sohail *et al*. 2011 |
|  |  |  |
| Female Japanese quails  At d 35 for 12 wk were exposed to:  1. Cont. at 22°C  2. CyCHS at 34°C for 8 h/d | **Liver:** HSP70 levels, MDA ↑; CAT, SOD, GSH-Px activities ↓ | Sahin *et al*. 2013 |
| Female Xuefeng black-boned chickens  At d 42 for 15 d were exposed to:   1. Cont. at 24±2°C 2. CyCHS at 37±2°C for 8 h/d followed by 24±2°C | **Serum:** MDA ↑; GSH, GSH-Px, SOD and CAT activity ↓  **Bursa of Fabricius**: HSP27, HSP70 and HSP90 mRNA levels ↑  **Thymus**: HSP27and HSP90 mRNA levels ↓; HSP70 mRNA levels ↑  **Spleen**: HSP27, HSP70 and HSP90 mRNA levels ↑ | Liu *et al*. 2014 |
| MDA, malondialdehyde; RH, relative humidity; CAT, catalase; SOD, superoxide dismutase; GSH, glutathione; GSH-Px, glutathione peroxidase; GR, glutathione reductase; H_2_O_2_, hydrogen peroxide; 3HADH, 3-hydroxylacyl CoA dehydrogenase; CCO, cytochrome C oxidase; CS, citrate synthase; avUCP, avian uncoupling proteins; FCCP, carbonyl cyanide p-trifluoromethoxyphenyl hydrazone (an uncoupler for repiratory chain); Nrf2, nuclear factor erythroid 2–related factor 2; GSSG, glutathione disulfide; HSP, heat shock protein | | |
